# Impact of Polyphenols on Inflammatory and Oxidative Stress Factors in Diabetes Mellitus: Nutritional Antioxidants and Their Application in Improving Antidiabetic Therapy

**DOI:** 10.3390/biom13091402

**Published:** 2023-09-17

**Authors:** Michal Krawczyk, Izabela Burzynska-Pedziwiatr, Lucyna A. Wozniak, Malgorzata Bukowiecka-Matusiak

**Affiliations:** Department of Structural Biology, Medical University of Lodz, 90-419 Lodz, Poland; michal.s.krawczyk@gmail.com (M.K.); izabela.burzynska-pedziwiatr@umed.lodz.pl (I.B.-P.); lucyna.wozniak@umed.lodz.pl (L.A.W.)

**Keywords:** oxidative stress, inflammation, polyphenols, diabetes

## Abstract

Diabetes mellitus is a chronic metabolic disorder characterized by hyperglycaemia and oxidative stress. Oxidative stress plays a crucial role in the development and progression of diabetes and its complications. Nutritional antioxidants derived from dietary sources have gained significant attention due to their potential to improve antidiabetic therapy. This review will delve into the world of polyphenols, investigating their origins in plants, metabolism in the human body, and relevance to the antioxidant mechanism in the context of improving antidiabetic therapy by attenuating oxidative stress, improving insulin sensitivity, and preserving β-cell function. The potential mechanisms of, clinical evidence for, and future perspectives on nutritional antioxidants as adjuvant therapy in diabetes management are discussed.

## 1. Introduction

Diabetes mellitus (DM) is a global health concern with increasing prevalence. It is a chronic metabolic disorder characterized by high blood glucose levels (hyperglycaemia) due to inadequate insulin production, impaired insulin action, or a combination of these [1]. Diabetes is classified into two types: type 1 and type 2. Diabetes can appear during pregnancy, under other conditions, such as drug or chemical toxicity, genetic disorders, endocrinopathies, and insulin receptor disorders, and in association with pancreatic exocrine diseases.

Hyperglycaemia occurs in type 1 diabetes mellitus (T1DM) due to a complex disease process in which genetic and environmental factors cause an autoimmune response that has yet to be fully understood [2]. The pancreatic cells within the islets of Langerhans are destroyed during this process, resulting in individuals with this condition relying primarily on exogenous insulin administration for survival. However, a subset has significant residual C-peptide production [3]. Type 1 diabetes is usually diagnosed in children and young adults, although it can develop at any age. It accounts for about 5–10% of all diabetes cases. Indeed, several major genetic determinants of T1DM account for only 40–50% of the familial clustering of this disorder [4]. Furthermore, there is a 6% annual increase in the risk of developing T1DM in developed countries, which remains unexplained but is thought to be caused by environmental triggers [5].

Type 2 diabetes mellitus (T2DM), also known as non-insulin-dependent diabetes or adult-onset diabetes, is characterized by insulin resistance and inadequate insulin secretion. In this condition, cells become less responsive to insulin, leading to elevated blood sugar levels. Type 2 diabetes is the most common form of diabetes, accounting for approximately 90–95% of all cases. It often develops in adults but has become more prevalent in children and adolescents due to lifestyle changes and obesity [6]. The link between the obesity and DM is related to adiposopathy, meaning the dysfunction in hormonal activity of adipose tissue promotes chronic inflammation, dysregulated glucose homeostasis, and impaired adipogenesis, leading to the accumulation of ectopic fat and insulin resistance [7]. Peripheral insulin resistance and the compensatory hypersecretion of insulin from pancreatic islets may precede a decline in islet secretory function in this form of the disease. Skeletal muscle, liver, and adipose tissue have the most prominently reduced insulin sensitivity due to these sites’ specific glucose uptake and metabolism requirements. However, it is increasingly thought that in most subjects, a relative decrease in insulin secretion is the final event that leads to hyperglycaemia [8]. The rise in the incidence of type 2 diabetes, particularly in developing countries, corresponds to the trend of urbanization and lifestyle changes, perhaps most notably a “Western-style” diet with resulting obesity. This suggests that environmental factors play an essential role in this disease’s pathogenesis, which also has a strong genetic component. It is still unlikely that genetic factors or aging alone can explain the dramatic rise in type 2 diabetes prevalence. It is unclear how increased caloric and dietary fat intake, reduced exercise, and an associated increase in body weight lead to type 2 diabetes.

Gestational diabetes mellitus (GDM) affects 2–10% of all pregnant women, depending on the type of applied diagnostic tests and investigated population [9]. There has been rapid growth recently in the incidence of GDM, both throughout the world and in Poland, caused by the obesity epidemic, the increase in mothers’ ages, and a lack of physical activity [10]. Gestational diabetes leads to numerous severe complications in both mothers and their children [11]. Considering mothers, GDM is a significant risk factor for developing type 2 diabetes later in life; it is estimated that approx. 20–30% of women with gestational diabetes develop type 2 diabetes within 10 years of having a baby. The risk of T2DM is almost 10 times higher in women diagnosed with GDM than in women whose pregnancy is proceeding correctly [12]. In turn, children of women with GDM are at higher risk of developing macrosomia, neonatal hypoglycaemia, and jaundice complications. At a later age, the consequences may relate to the onset of metabolic syndrome, obesity, and diabetes [13].

In 2014, there were 422 million patients who were affected by diabetes, nearly twice as many as in 1980 [9]. According to projections, there will be 578 million diabetic patients by 2030 and 700 million by 2045 [14]. The disease reduces both a person’s quality of life and life expectancy. It imposes a significant economic burden on the health care system globally and affects the lives of patients and their families.

Oxidative stress, resulting from an imbalance between reactive oxygen species (ROS) production and antioxidant defence, contributes to the pathogenesis of diabetes and its complications [15]. Nutritional antioxidants in various dietary sources have shown promise in reducing oxidative stress and improving glycaemic control in individuals with diabetes. These antioxidants can exert their effects by neutralizing ROS, modulating intracellular signalling pathways, and enhancing the antioxidant defence system [16]. An antioxidant-rich diet has been found to decrease oxidative markers and improve insulin sensitivity in T2DM individuals [17],which also has been supported by the results obtained by van der Schaft and coworkers [18].

The enzymatic antioxidant defence system is pivotal in neutralizing ROS and maintaining cellular redox homeostasis. Critical components of this system include superoxide dismutase (SOD), catalase, and glutathione peroxidase, along with sirtuins and peroxisome proliferator-activated receptor gamma (PPAR-gamma) [19]. The study conducted by Guzik et al. found that diabetes-related vessels produce significantly more superoxide from two main sources: first, the activity and protein levels of the vascular NAD(P)H oxidase system are increased; second, in diabetic vessels, the endothelium is a net contributor to the total vascular superoxide release rather than superoxide scavenging by NO production. This increased endothelial superoxide production appears to be caused by dysfunctional endothelial NO synthase (eNOS), which is mediated by tetrahydrobiopterin (BH_4_) availability. Finally, Protein kinase C (PKC) signaling appears to be involved in these changes, at least in part [20].

A diverse group of organic compounds called polyphenols is abundant in plants. They have attracted much interest because of their potential health advantages, especially as antioxidants.

Numerous plant-based food sources, such as fruits, vegetables, whole grains, legumes, nuts, and seeds, are high in polyphenols. Berries (such as blueberries, strawberries, and raspberries), citrus fruits, apples, grapes, green tea, cocoa, and spices such as turmeric and cinnamon are notable sources of polyphenols. The specific polyphenol content varies among plant sources. Therefore, it is beneficial to incorporate a diverse range of fruits, vegetables, and other plant-based foods into one’s diet [21].

This review outlines the recent progress in polyphenols’ application as an anti-inflammatory and antioxidant factor in diabetes mellitus with particular emphasis on molecular mechanisms.

## 2. Damaging Effects Mediated by Oxygen Free Radicals

The term “reactive oxygen species” (ROS) encompasses molecules such as free radicals, neutral atoms, molecules, and ions [22,23]. Free radicals are molecules with one or more unpaired electrons on the atomic or molecular orbitals, and they are natural byproducts of oxygen metabolism in all aerobic organisms. Oxygen is the precursor of radicals in aerobic organisms. The molecule in the ground state is the diradical (•O=O•) consisting of two unpaired electrons on the two anti-bonding orbitals p*2p. The oxygen molecule can be excited with the change of spin of one electron on orbital p*2p, forming a singlet oxygen molecule (^1^O_2_). This molecule is highly reactive, can directly induce lipid peroxidation, DNA (particularly guanine), and protein oxidation (particularly histidine, tyrosine, and tryptophan), and participates in the synthesis of other ROS [24]. Free radicals are highly reactive species that can induce chain reactions resulting in new free radicals’ synthesis. Singlet oxygen (^1^[O_2_]), hydrogen peroxide (H_2_O_2_), peroxide ion (O_2_^−^), and hypochlorous acid (HOCl) are nonradical ROS. ROS also comprise oxygen radicals, including the superoxide anion (O_2_•^−^), hydroxyl radical (HO•), hydroxide radical (HO_2_•), peroxide radical (ROO•), and alkoxyl radical (RO•) [25,26,27].

**Figure 1:**
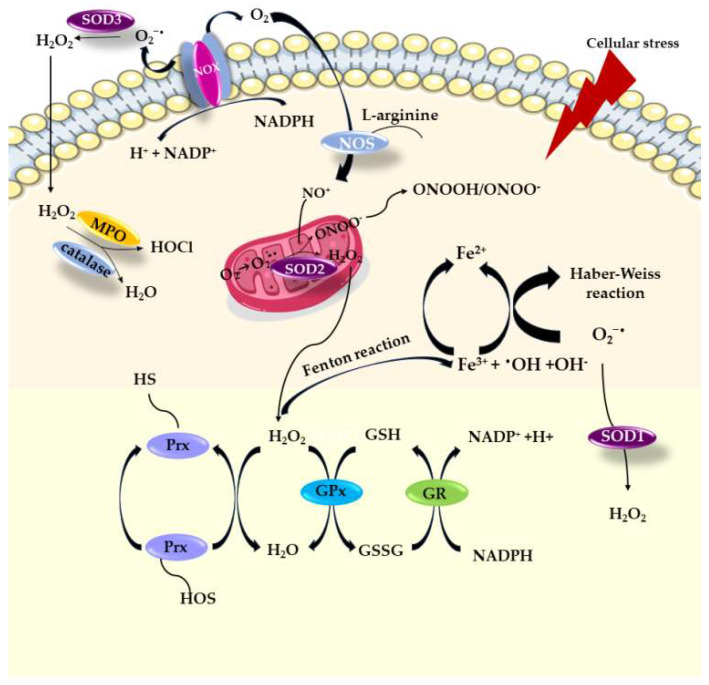
Sources of reactive oxygen species in the cell.

ROS are formed in living organisms as a result of the action of various external environmental factors, including ionizing and ultraviolet radiation, ultrasound, or cigarette smoke (exogenous sources), as well as numerous intracellular biochemical processes occurring in mitochondria, peroxisomes, microsomes, or phagocytic leukocytes (endogenous sources) [27,28] (Figure 1).

The primary sources of ROS are mitochondria in the mitochondrial electron transport chain, where ATP, as a result of oxidative phosphorylation, is produced. About 1–3% of all electrons in the transport chain are “leaking” to generate superoxide which can be further reduced to HO^•^ and H_2_O_2_ instead of contributing to the reduction of oxygen to water. The mitochondrial respiratory chain (RC) is a significant site of ROS production in the cell. Therefore, it has been suggested that mitochondria are the prime targets for oxidative damage [25]. Several alternative pathways also lead to their formation: NADPH-oxidase (NOX), immune reactions, xanthine oxidase, arachidonic acid metabolism, etc. [29].

ROS are poisonous and pathogenic at uncontrolled high concentrations, but at physiological concentrations, ROS are mediators and regulators of many critical cellular processes, such as proliferation, differentiation, and apoptosis [30,31]. ROS can thus play a significant physiological role as secondary signalling messengers through protein kinases, transcription factors, and proinflammatory factors’ genomic expression [32]. It has been found that some growth factors can induce ROS production in non-phagocytic cells by interacting with specific receptor proteins [33].

When ROS overwhelm the cellular antioxidant defence system, whether through an increase in ROS concentration or a decrease in the cellular antioxidant capacity, oxidative stress occurs. Oxidative stress results in direct or indirect ROS-mediated damage, including nucleic acids (DNA or RNA) [34], the oxidation of proteins [35], and the lipid peroxidation of polyunsaturated fatty acids (such as membrane phospholipids) [36]. These changes have been identified in carcinogenesis, neurodegeneration, atherosclerosis, diabetes, and ageing [37]. However, the involvement of ROS in the pathogenesis of diseases is not confined to macromolecular damage. There is increasing evidence that ROS signalling contributes to diseases.

### 2.1. Oxidative Damages of DNA

To date, more than 100 products have been identified from the oxidation of DNA. ROS reactions with DNA lead to a multitude of oxidative damage to macromolecules, which comprise (a) single- or double-strand breaks of DNA; (b) the structural modification of purine and pyrimidine bases; (c) the formation of DNA-protein binding and the formation of various types of adducts; (d) the introduction of basic sites; and (e) the cross-linking between DNA chains [38,39,40]. The consequences of damage to DNA may include the inhibition of replication and transcription or the presence of mutations leading to genomic instability. All these changes lead to disturbances in the normal functions of cells and the development of many diseases, including cancer and ageing [23,41].

**Figure 2:**
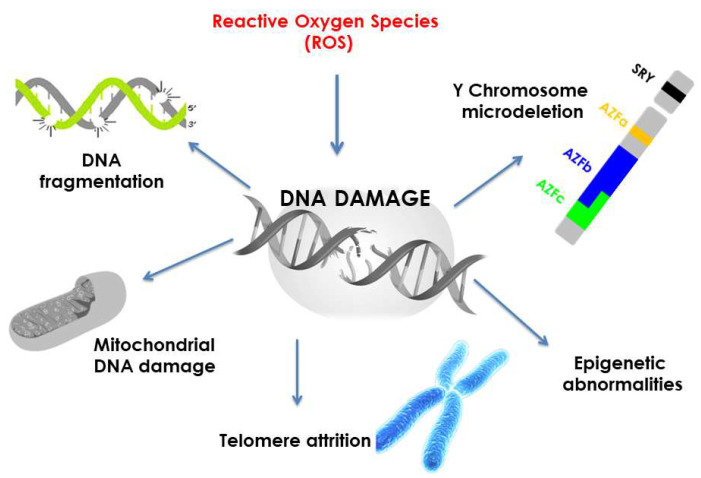
Types of ROS-mediated DNA damage based on Bui et al. [44].

Oxidative DNA damage encompasses mainly physicochemical changes in DNA, which can affect the interpretation and transmission of genetic information [42,43]. ROS (mainly HO•) in DNA can react with the nucleobases and deoxyribose, triggering significant oxidative reactions. This can lead to mutations, carcinogenesis, apoptosis, necrosis, and hereditary diseases [43]. DNA breaks occur due to nucleosome fragmentation (fundamental structures for the organization of DNA within chromosomes), thus initiating complications in forming DNA within chromatin. Chromatin plays an essential role in regulating gene transcription [44], and accordingly, modifications in its functional properties may result in errors leading to mutagenesis (Figure 2).

The hydroxyl radical adds to purines, giving rise to C4-OH-, C5-OH-, and C8-OH-adduct radicals, while the addition to the pyrimidine leads to the formation of adducts at the C5-OH and C6-OH positions. 8-hydroxyguanine (8-OH-dG) has been the most extensively studied one so far. It is relatively quickly formed and is both mutagenic and carcinogenic. It is a promising biomarker of an organism’s oxidative stress and a potential carcinogenesis biomarker [45]. It has been demonstrated that 8-OH-dG induces transversions of GC → TA, whose presence has been identified in several mutant oncogenes (e.g., the DNA mismatch repair protein—MSH2) and tumour suppressor genes (e.g., P53, BRCA1, APC) [46]. Other known products of oxidative DNA damage, which are also highly mutagenic, include 8-oxo-adenine, 5-hydroxyuracyl, thymine glycol, and uracil glycol [47].

Mitochondrial DNA (mtDNA) is particularly susceptible to oxidative damage since mitochondria have limited repair mechanisms through nucleotide excision. Moreover, mtDNA is not protected by histone proteins. The results of many studies have confirmed respiratory chain dysfunction in many cancer cells due to mutations in the genes encoding its enzymatic components [22,45].

DNA damage in cells leads to point mutations in particular regulatory genes that may activate oncogenes and inactivate tumour suppressor genes, thus initiating and developing cancer. However, specific and general repair mechanisms can repair DNA base modifications. In the case of extending oxidative damage to DNA that repair mechanisms cannot cope with, the cell goes into the process of programmed death called apoptosis.

Increased levels of 8-oxo-dG in the tissues of diabetic rats and the urine of patients with type 1 and type 2 diabetes were observed [48] with the levels being significantly higher in patients with albuminuria or other diabetic complications [49]. In patients with impaired fasting glucose in the prediabetic state, their levels of 8-OHdG increased compared to those of normoglycemic patients [50]. An increased concentration of 8-Oxo-dG and TG in familial combined hyperlipidaemic (FCH) subjects was detected compared to that of healthy controls. 8-oxo-dG was positively correlated with insulin and triglycerides and negatively with high-density lipoprotein cholesterol in FCH subjects [51]. According to Simone et al.’s study, the hyperglycaemia-induced redox-dependent activation of protein kinase—Akt—augmented the phosphorylation of the tuberin protein and, as a consequence, also downregulated human 8-oxoguanine-DNA glycosylase 1 (hOGG1), an enzyme engaged in the DNA base excision repair pathway (BER) [49].

Some studies have directly correlated DNA damage and HbA1c in T2DM individuals. When Xavier et al. [52] investigated peripheral blood mononuclear cells, individuals with poor glycaemic control (HbA1c > 7%) revealed higher DNA damage indicator levels compared to those of normoglycemic (HbA1c < 7%) individuals. In leukocytes from controlled T2DM patients, an elevated level of DNA damages was observed in comparison to that of healthy individuals [53,54]. However a positive correlation between antioxidant capacity and glucose or HbA1c levels in poorly controlled T2DM subjects was noted, which is consistent with the theory that their antioxidant capacity is improved to compensate for the excess of free radical species [54]. Other studies have examined blood lymphocytes obtained from T2DM patients, which exhibit increased DNA oxidative damage, a more heightened receptivity for mutagens, and insufficient DNA repair systems, further contributing to the development and progression of T2DM and an increased risk of cancer in those individuals [55].

Some literature data indicate a correlation between DNA damage and the risk of GDM development; however, the results are inconsistent. Patients with GDM or mild gestational hyperglycaemia may have higher oxidative DNA damage [56,57].

### 2.2. Lipid Peroxidation

Polyunsaturated fatty acids (PUFAs) present in membrane phospholipids are very susceptible to oxidation by ROS [58]. ROS, particularly hydroxyl and peroxyl radicals, can react with PUFAs and initiate lipid peroxidation chain reactions [59,60].

The first step of lipid peroxidation is an initiation reaction, which starts with the hydrogen abstraction from the methylene group of polyunsaturated fatty acids (in particular, linoleic and arachidonic acid), and, as a result, the alkyl radical (R^•^) is formed. In the next propagation step, a radical R^•^ reacts with oxygen to form peroxyl radicals (ROO^•^) capable of abstracting a hydrogen atom from another polyunsaturated fatty acid and thus starting a chain reaction. As a result, fatty acid peroxides (ROOH) and another radical, R^•^, are formed. The free radical chain reaction propagates until two free radicals conjugate each other to terminate the chain. The reaction can also terminate in the presence of a chain-breaking antioxidant such as vitamin C or vitamin E (α-tocopherol).

Lipid peroxidation products may be endoperoxides, epoxides, aldehydes, or dimers of fatty acids. Malondialdehyde (MDA) is the best-known end product of lipid peroxidation. It has been found that MDA has mutagenic properties by forming DNA adducts in bacterial and mammalian cells and is carcinogenic in rats [61]. In addition to MDA, in the process of lipid peroxidation, other α, β-unsaturated aldehydes and hydroxy aldehydes, including 4-hydroxyalkenals, 2-alkenals, hepta-2,4-dienal, 5-hydroxyoktanal, and 4-hydroxy-2-nonenal (HNE) are generated. HNE is mutagenic and highly toxic. MDA and HNE are good oxidative stress markers, and their presence has been confirmed, i.a., in cardiovascular diseases, diabetes, and Parkinson’s and Alzheimer’s diseases [62]. 4-HNE, which reacts with cellular proteins and DNA, giving stable adducts, is a signalling molecule that affects the regulation of several stress-sensitive transcription factors, such as Nrf2, activating protein-1 (AP-1), NF-κB, and peroxisome proliferator-activated receptors. Moreover, it is also involved in activating some stress response pathways, such as MAPK, epidermal growth factor receptor/Akt, and PKC pathways [59].

ROS-induced lipid peroxidation contributes to reactive carbonyl species (RCS) formation which plays a vital role in the development of various diseases, for example, diabetes. RCS may have cytotoxic and genotoxic properties depending on their concentration at the tissue or the systemic level. The augmented concentration of 4-HNE in serum, plasma, blood, urine, cells, and tissues may be linked to diabetic complications [63]. Many studies on type 1 and type 2 diabetes from various tissues for 4-HNE levels confirmed this concept [64,65]. In type 1 diabetic mice, about a 2.5-fold higher level of 4-HNE was detected compared to that of the controls [65].

A higher level of 4-HNE-modified proteins in the pancreatic beta cells of aged type 2 diabetic rats was observed compared to that of the controls, correlated with an increase in the fibrosis of pancreatic islets [66]. Toyokuni et al. revealed significantly higher levels of 4-HNE-modified albumin in the serum of type 2 diabetes patients [64].

MDA may be a promising biomarker in T2DM. Uncontrolled T2DM patients with an HbA1c level >7% exhibited significantly higher MDA levels than the control group. However, there was no statistically significant difference between T2DM-controlled patients (HbA1c level < 7%) and the control group. Moreover, a significant positive correlation between the MDA concentration and lipid parameters (except HDL-C) was observed, which may indicate the coexistence of atherogenic risk factors and oxidative stress [67]. Furthermore, the MDA levels were elevated compared to those of normoglycemic controls in T2DM patients without complications and with retinopathy [68]. Desco et al. also reported higher levels of plasma MDA in type 1 DM patients [69].

### 2.3. Oxidations of Proteins

The mediators of oxidative damage to proteins are HO^•^ and, to a lesser extent, O^•^_2_^−^ and H_2_O_2_. The reaction of HO^•^ with the protein backbone starts with the abstraction of the hydrogen atom from the α carbon atom of an amino acid, which leads to the creation of an alkyl radical which then reacts with oxygen to form a radical alkyl peroxide [70]. This product is then transformed into alkylperoxide. In the next step, alkylperoxide is converted into an alkoxyl radical which may be converted into a hydroxyl derivative of an amino acid or lead to the breaking of the polypeptide chain. In the case of oxygen deficiency, the formation of radical alkylperoxide is demanding, and alkyl radicals react with each other, forming cross-links between polypeptide chains.

Thiol groups of cysteine and methionine are particularly susceptible to ROS attacks resulting in the formation of both intramolecular (P1-SS-P1) and intermolecular (P1-SS-P2) disulfide bridges and oxidation products containing sulfenic group sulfinate or sulfonate, metionylosulfoxides, or metionylosulfonyl [71].

The aromatic amino acid residues are also highly susceptible to ROS attacks. It has been found that the oxidation of tyrosine residues formed 3,4- dihydroxyphenylalanine or 2,5-tyrosine and the oxidation of tryptophan residues formed formylkynurinine and kynurenine [72]. The markers of oxidative damage to proteins are carbonyl derivatives formed by the oxidation of amino acids containing free amino-, amido- or hydroxyl- groups by ROS. Carbonyl derivatives may react with the free amino groups of lysine residues, leading to cross-link formation in the same or another protein molecule. Modified and improperly folded protein chains are directed to the proteolytic pathways and eliminated through proteasome ubiquitination [72].

**Figure 3:**
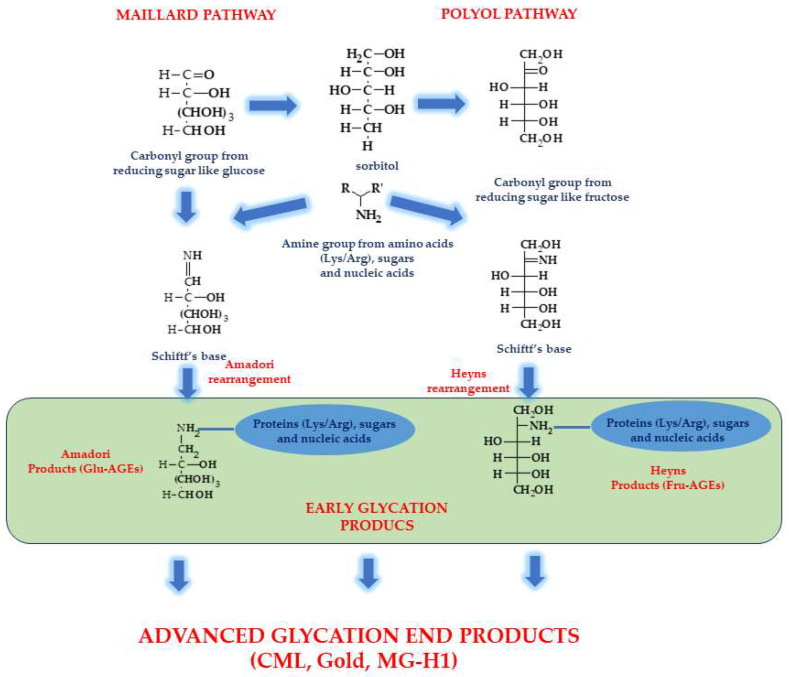
Pathways leading to advanced glycation end products (AGEs). Glu-AGE—glucose-derived AGE; Fru-AGE—fructose-derived AGE.

Advanced glycation end products (AGEs) are diverse compounds that may be formed in non-enzymatic reactions between glucose or other saccharides and proteins or lipids. Environmental factors, including cigarette smoke, high levels of refined and simple carbohydrates, hypercaloric diets, foods cooked at a high temperature, and a sedentary lifestyle, can initiate the production of AGEs and consequently damage cell lipids and proteins [67,73]. ROS may also catalyse the formation of AGEs and advance lipooxidation end products (ALEs). AGEs are synthesized in the non-enzymatic Maillard reaction of nonreducing sugars and amino groups in proteins, lipids, and nucleic acids, resulting in nonstable Schiff bases that undergo the Amadori rearrangement, leading to the formation of stable ketamine. These compounds may be transformed into more stable products (with peptides or proteins to form protein cross-links) in a reversible reaction. An alternative to AGEs is the polyol pathway in which the Heyns product is created, followed by Heyns rearrangement [74]. The AGEs of these pathways belong to the following groups: fructose-derived AGEs (Fru-AGE) and glucose-derived AGEs (Glu-AGE) (Figure 3)[75].

These compounds may also participate in oxidation, dehydration, or polymerization reactions to result in numerous other AGEs [76,77].

The oxidation of proteins is associated with the loss of biological activity and an increased susceptibility to aggregation and proteolysis. AGEs are involved in cell toxicity. AGEs can play a central role in the pathophysiology of different diseases, for example, diabetes. The AGE-induced pathophysiology of diabetes mellitus encompasses two mechanisms. AGEs may act directly by trapping and cross-linking proteins or indirectly by binding to the cell surface receptor.

AGEs can interact and modulate cell signalling through binding to various receptors, for example, Toll-like receptors, scavenger receptors, G-protein-coupled receptors, and pattern recognition receptors (RAGEs). The latter are specific receptors for AGEs, and, in homeostasis, they are expressed at basal levels.

The accumulation of a high amount of sugar compounds may contribute to increased levels of AGEs called glycotoxins and their interactions with human amino acids, peptides, and proteins. There is a hypothesis that carbohydrates from food can affect internal glucose levels and impair glucose pathways in tissues, which, consequently, can strongly and negatively influence human health [78]. Under pathological conditions or chronic inflammation, such as diabetes mellitus (DM), cardiovascular disease, Alzheimer’s disease, cancer, and natural ageing, their expression is elevated. It has been reported that in diabetes, both ROS and AGE concentrations are increased [79]. Elevated concentrations of AGEs in the bloodstream, a consequence of hyperglycaemia in diabetes, may induce a signalling cascade through RAGE. This activation involves a variety of downstream effectors, including mitogen-activated protein kinase (MAPK), p38, stress-activated protein kinase/c-Jun N-terminal kinase (SAPK/JNK), Ras-mediated extracellular signal-regulated kinase (ERK1/2), and Janus kinase signal transducer and activator of transcription (JAK/STAT) pathway which will sequentially lead to sustained activation transcription factors, such as NF-κB, STAT3, HIF-1α, and AP-1 [74,80,81]. Mounting evidence suggests that AGEs/RAGE-induced signalling pathways encompassing NF-κB activation, inflammation, and ROS formation are directly linked to the pathogenesis of insulin resistance by increased IRS-1 serine phosphorylation and degradation, therefore blocking the insulin signalling pathway [82]. Hyperglycaemia resulting from elevated AGE levels in the pancreas may have a toxic effect on beta cells by triggering inflammatory cascades and oxidative stress [83,84]. This effect can be observed as upregulated RAGE expression in pancreatic islets [85]. It also confirmed that AGEs may be suitable biomarkers in gestational diabetes mellitus (GDM) by increasing the concentrations of TNF-α (tumour necrosis factor-alpha) and hs-CRP (high-sensitivity C-reactive protein), which are responsible for inflammatory reactions [86].

## 3. The Role of Oxidative Stress and Inflammation in Aetiology of Diabetes Mellitus

Pancreatic β-cell dysfunction and insulin resistance in hepatocytes, myocytes, and adipocytes significantly contribute to T2DM pathogenesis. The cellular and molecular mechanisms underlying these abnormalities are not fully understood. The imbalance between the production and elimination of reactive oxygen species (ROS) causes oxidative stress (OS), which stems from chronic low-grade inflammation and chronic hyperglycaemia resulting from insulin resistance, leading to abnormal cytokine overproduction and the activation of inflammatory signalling pathways in the above-mentioned insulin-sensitive tissues [87]. Hyperglycaemia linked to insulin resistance is the major pathogenetic factor of T2DM. Numerous pre-clinical and clinical studies investigate the influence of increased glycaemia on redox homeostasis and inflammatory responses. OS plays a pivotal role in the pathophysiology of diabetic complications related to lipid peroxidation, DNA damage, and mitochondrial dysfunction. Its involvement is visible in many other pathological conditions and age-related disorders. Ageing-related disorders are defined as the progressive loss of function in tissues subjected to OS and many other mechanisms [88]. The oxidative stress theory by many researchers is considered the primary background of ageing and age-related complications. Thus, maintaining the proper balance of the redox state is essential in oxidative stress-induced complications and insulin resistance prevention [89].

**Figure 4:**
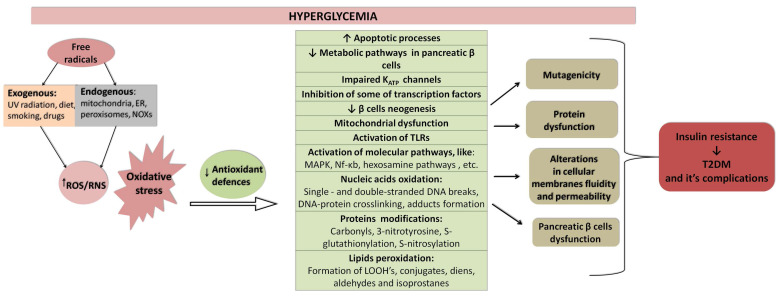
Molecular mechanisms correlating oxidative stress, its outcomes, and hyperglycaemia, which lead to insulin resistance and T2DM development. Abbreviations: TLRs—Toll-like receptors; Nf-κb—nuclear factor kappa b; MAPK—mitogen-activated kinases. Legend: ↓—decrease, ↑—increase.

Some of the mechanisms described below underlying the development of T2DM and its complications in correlation to OS are presented in Figure 4. This chapter provides evidence on how increased glucose and insulin resistance affect ROS overproduction and the antioxidant defence system, leading to OS and immune system activation in diabetic individuals (Figure 4).

Prediabetes, an elevated blood glucose level not reaching the diabetes criteria (the range from 100 to 125 g/dL (5.6 mmol/L to 6.9 mmol/L) according to ADA criteria) [1], can predispose patients to T2DM due to hyperglycaemia-related OS, underlying ROS overproduction, and increased inflammation markers. Prolonged inflammation and OS overload can lead to impaired insulin secretion, insulin resistance, and further T2DM development [90]. Some OS- and inflammation-related changes are already present in the prediabetes phase; ongoing and progressing prediabetes-related hyperglycaemia enhances these alterations. Evolving OS leads to muscle insulin resistance, impaired pancreatic cell function, and insulin secretion. Some studies have revealed that using nicotinamide adenine dinucleotide phosphate (NADPH) oxidase inhibitors (Apocynin) causes the reduction of OS in a prediabetes state [91]. It has been shown that insulin resistance is associated with OS in non-diabetic individuals and those with increased risk factors for DM development, such as obesity or impaired fasting glucose. The involvement also of the gastrointestinal tract in glucose homeostasis is nowadays emphasised as an important factor in DM development and progression [92] which is why many ongoing studies investigate the influence of a polyphenol-rich diet and the incorporation of polyphenols into the gastrointestinal tract in other forms as well as their beneficial outcomes on DM management and treatment in the context of OS [93,94]. The Framingham Offspring Study on the association of oxidative stress, insulin resistance, and diabetes risk phenotypes revealed a positive correlation between insulin resistance and urinary 8-epi-prostaglandin F2α (8-epi-PGF2α),- an OS marker [95].

Lipid peroxidation, DNA damage, and mitochondrial dysfunction are some of the examples of processes by which the OS executes a pivotal role in the pathophysiology of various complications of diabetes. Several experimental studies have proven that increased mitochondrial ROS production is associated with high glucose levels in leukocytes, endothelial cells, and adipocytes. Pancreatic β-cells are prone to glucose toxicity due to chronic OS in DM caused by the deficient expression of antioxidant enzymes compared to other tissues [96]. It has been proposed that excessive ROS production may cause the impairment of β-cells by the decreased expression and DNA-binding activity of some tissue-specific transcriptional factors, precisely pancreas duodenum homeobox-1 and V-maf musculoaponeurotic fibrosarcoma oncogene homolog A. This phenomenon causes reduced insulin gene expression, content, and secretion [97].

Acharya et al. [98] revealed the correlation between hyperglycaemia, oxidative stress, and β-cell dysfunction in newly diagnosed diabetic individuals when comparing the OS parameters at their time of diagnosis and eight weeks after antihyperglycemic treatment, thus finding a significantly increased OS at at the point of diagnosis with T2DM. Irrespective of the type of hypoglycaemic treatment used, lowered oxidative stress and improved β-cell function were observed.

Numerous ongoing and completed clinical trials have revealed the importance of anti-inflammatory constituents and their contribution to glucose homeostasis. A study investigating the glucose-lowering effect of Salsalate (a pro-drug of salicylate) demonstrated its effectiveness in lowering the HbA1c and fasting glucose levels of T2DM individuals [99].There is a growing body of evidence showing that low-grade chronic inflammation is one of the multiple factors underlying the pathophysiology of insulin resistance, DM, and its complications; furthermore, it is also clear that these processes might affect the insulin signalling transduction and beta cell function [61,100]. Janus kinase pathways (JNKs) induced by some cytokines further stimulate IRS-1 serine phosphorylation causing abnormalities in insulin signalling pathways [101]. Other inflammatory mediators, such as TNF-α and Nf-κb, have been reported to modulate IRS-1 serine phosphorylation and further affect the proper insulin signalling transduction.

In patients with T2DM, the increased activity of monocytes and macrophages and increased levels of proinflammatory mediators, such as CX3CL1 (fractalkine), CRP, TNF-α, IL-6 (interleukin-6), IL-1β, IL-18, MCP-1 (monocyte chemoattractant protein-1), resistin, PAI-1 (plasminogen activator inhibitor-1), E-selectin, and IFN-γ (interferon-gamma), have been reported, which indicates the potential benefits of incorporating agents of anti-inflammatory activity into therapeutic approaches in diabetes management. Additionally, Toll-like receptors (TLR) 2 and 4 were found to be suppressed in peripheral mononuclear cells in T2DM patients [102]. Guerrero-Romero et al. revealed a strong association between hyperglycaemia/poor glycaemic control and increased CRP levels in prediabetic and diabetic patients [103]. Besides the increased CRP levels, IL-6 was also found to be elevated in diabetic individuals [104]. However, it has been proven that an improvement in glycaemic control is accompanied by a reduction in the above-mentioned inflammatory markers and the TBARS (thiobarbituric acid-reactive substances) concentration—an OS marker [105].

The monocyte cell subset is essential among circulating immune cells considering diabetes’ pathophysiology due to the increased levels of monocyte proinflammatory cytokines measured in T2DM individuals [106]. Thus, human monocytes have become the subject of many in vitro studies, revealing the upregulation of critical inflammatory genes such as cytokines—TNF-α and IL-1β, their receptors, and chemokines—MCP-1 in high-glucose conditions of culture [107]. The NF-κB regulates many hyperglycaemia-induced genes; thus, this transcription factor’s importance in regulating high-glucose-induced immune responses has been broadly investigated. The CREB-binding protein (CBP) and p300, both displaying intrinsic HAT (histone acetyltransferase) activity, are coactivators for p65, the NF-κB component. The high-glucose-induced hyperacetylation of p65 and the inhibition of HDACs (histone deacetylases) have been reported to be stimulated by the CBP/p300, resulting in NF-κB activation and the further increased transcription of IL-6 and TNF-α in monocytes [108].The above-mentioned study by Yun et al. revealed curcumin’s ability to decrease monocyte hyperglycaemia-induced cytokine production, probably via epigenetic changes in NF-κB, proving the direct action of curcumin on the inflammatory response. Since then, curcumin, a polyphenolic compound, has become a valuable subject in antidiabetic approaches.

Gonzalez et al. [109] reported the decreased monocyte CD33 expression and increased TNF-α production by human monocytes under hyperglycaemic conditions, thus making CD33—a member of the family of sialic acid-binding, immunoglobulin-like lectins—a subject of many studies related to the hyperglycaemia-induced inflammatory response. Gonzalez et al., using α-tocopherol, reported the reversal of the above-mentioned alternations, suggesting that hyperglycaemia-induced oxidative stress might cause CD33 downregulation. Oxidative stress and inflammation are two significant abnormalities underlying the development and progression of T2DM. They are promoted by chronic hyperglycaemia, a key factor for DM, thus indicating the reciprocal nature of their correlation. All of the above-mentioned findings strongly suggest that antioxidative compounds can effectively improve disturbed insulin secretion and action, thus positively influencing human glucose and oxidative homeostasis.

### Enzymatic Antioxidant Defence System

Substances that can delay, prevent, or remove OS-related damages are classified as antioxidants and are categorized into two major groups: enzymatic and non-enzymatic antioxidants. Enzymatic antioxidants contain the enzymes that play a crucial role as the first line of defence against ROS, including superoxide dismutase (SOD; EC.1.15.1.1), catalase (CAT; EC 1.11.1.6), and glutathione peroxidase (GPx; EC 1.11.1.19). The nuclear erythroid 2-related factor-2 (Nrf2) is a transcription factor that regulates the expression of all the above-mentioned enzymes. Thus, Nrf2 maintains cellular redox balance by modulating the antioxidant response. In animal model studies, the beneficial effects of Nrf2 action on β-cell dysfunction and insulin resistance have been revealed, as well as on the development of diabetes-related micro- and macrovascular complications [110].

SOD is an enzyme that catalyses superoxide radicals’ dismutation into hydrogen peroxide and molecular oxygen. It exists in different isoforms, including copper-zinc SOD (SOD1) which is present in the cytosol, manganese SOD (SOD2) which is found in the mitochondrial matrix, and extracellular SOD (SOD3). These isoforms are distributed in different cellular compartments and exhibit distinct regulatory mechanisms. SODs are critical in scavenging superoxide radicals and preventing their conversion into more reactive species [111].

Catalase is a heme-containing enzyme in peroxisomes that facilitates the decomposition of hydrogen peroxide into water and molecular oxygen. Catalase efficiently detoxifies hydrogen peroxide, thereby protecting cells from oxidative damage. Its activity is tightly regulated through transcriptional, post-transcriptional, and post-translational mechanisms. It is present in most cells, tissues, and organs and, at higher levels, in the liver and erythrocytes [112].

**Figure 5:**
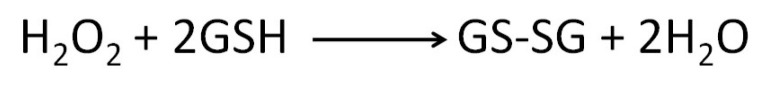
General mechanism for obtaining glutathione disulphide. GSH—glutathione; GS-SG—glutathione disulphide.

Glutathione peroxidases are a family of selenium-dependent enzymes that catalyse the reduction of hydrogen peroxide and lipid hydroperoxides using glutathione as a cofactor. These enzymes play a crucial role in maintaining the cellular redox balance and protecting against oxidative damage. Different isoforms of glutathione peroxidase are expressed in various tissues and exhibit distinct substrate specificities. Free thiol groups are oxidized to disulphide bonds during the process shown below, and reduced glutathione serves as an efficient electron donor [30] (Figure 5).

Glutathione is a tripeptide (γ-L-glutamyl-L-cysteinylglycine) present in millimolar concentrations in cells. It acts as a major intracellular antioxidant and participates in redox reactions by working as a reducing agent and a cofactor for enzymes such as glutathione peroxidase. Glutathione levels are tightly regulated, and alterations in its concentration have been associated with oxidative-stress-related disorders [112].

## 4. Polyphenols as Nutritional Antioxidants

Like other secondary metabolites, polyphenols serve as plants’ first line of defence. They are possibly the most significant non-nutrient bioactive groups in the human diet because plants constitute a primary nutritional component in human diets, and phenolics are more numerous than other phytochemicals in plant diets [113]. Among the several possible health advantages of dietary polyphenols, their capacity to inhibit oxidation stands out. They have consistently been demonstrated as powerful antioxidants, preventing oxidative damage and reducing inflammation. A diet high in phenolic-rich fruits, vegetables, and whole grains lowers one’s risk of cancer, cardiovascular disease, chronic inflammation, and metabolic disorders [21]. The mechanisms of phenolic compounds’ antioxidant and anti-inflammatory properties are linked with their capacity to scavenge free radicals, restore antioxidant enzyme activity, and regulate cytokine-induced inflammation.

In vitro, dietary phenolics are potent antioxidants, capable of neutralizing free radicals by donating an electron or a hydrogen atom to a wide range of reactive oxygen, nitrogen, and chlorine species, ^●^, including O_2_^●−^, OH peroxyl radicals RO_2_^●^, hypochlorous acid (HOCl), and peroxynitrous acid (ONOOH) [114]. As efficient radical scavengers, polyphenols disrupt the propagation stage of the lipid autoxidation chain reactions or function as metal chelators to convert hydroperoxides or metal prooxidants into stable molecules. As metal chelators, phenolic compounds can directly block Fe^3+^‘s reduction by lowering the formation of reactive OH in the Fenton reaction [115]. Although phenolic acids and flavonoids have excellent radical scavenging action, their metal chelating potential and reducing power might differ depending on their structural characteristics.

Based on their chemical structure, the plant-derived antioxidants are classified as carotenoids, phenolics, alkaloids, nitrogen-containing compounds, and organosulfur compounds, along with the sterols, terpenes, fibre, and organoselenium compounds which are also distinguished in such a classification [113]. Flavonoids (such as flavonols, flavanols, flavones, and anthocyanins), phenolic acids (ellagic acid, caffeic acid), stilbenes (resveratrol), and lignans are just a few of the numerous substances that fall under the category of polyphenols. Every type of polyphenol has distinct antioxidant qualities and health advantages [116]. Synergistic interactions between polyphenols frequently result in combined effects that are more significant than the sum of their individual effects. These findings suggest that eating foods high in polyphenols may offer better antioxidant protection than taking supplements of isolated polyphenols [117].

Polyphenols have anti-inflammatory effects in addition to antioxidant ones. By lowering the production of proinflammatory molecules and fostering a more balanced immune response, they can help modulate inflammatory pathways. Polyphenols may aid in the prevention and treatment of inflammatory diseases by reducing chronic inflammation [118].

Additionally, polyphenols can affect the cellular signalling networks that control inflammation and oxidative stress. Specific enzymes, such as Nrf2 (nuclear factor erythroid 2-related factor 2), also govern antioxidant defence. Polyphenols can activate these enzymes, increasing the body’s natural antioxidant capacity by modulating these pathways [119]. In addition to neutralizing free radicals in the aqueous environment, polyphenols shield lipids from oxidation. Lipid peroxidation can produce dangerous byproducts that harm cell membranes and exacerbate several diseases. The chain reaction of lipid peroxidation can be stopped by polyphenols, maintaining the structure and functionality of cellular membranes [120].

It is of high importance to also mention that numerous researchers are now making efforts to develop potentially beneficial and applicable synthetic derivatives of naturally occurring polyphenolic compounds that could be used as subjects for antidiabetic approaches. Compounds such as iminosugars and other sugar derivatives are some examples of subjects of ongoing studies and are being considered as promising for their potential applications as antidiabetic agents obtained by the chemical modification of plant-derived stem compounds [121].

## 5. Polyphenols Present in the Diet and Incidence of Various Diseases

Some studies indicate that the presence of polyphenols in one’s diet can reduce the risk of developing diseases, such as cardiovascular diseases, some types of cancer, and neurodegenerative diseases [122,123]. In a randomized double-blind placebo-controlled trial study, after supplementation with polyphenolic extract (Hibiscus sabdariffa and Lippia citriodora) for over 84 days, type 1 hypertensive patients of both sexes exhibited a decrease in daytime systolic blood pressure (SBP) in comparison to a placebo group. However, their diastolic blood pressure remained at similar values as the baseline. The mechanism by which the polyphenol extracts decreased their blood pressure may be related to the modulation of different metabolic pathways and the activation of the AMPK pathway favouring lipolysis and, therefore, fat loss. Polyphenols present in HS and LC extracts may possess vasodilatory properties and the capacity to inhibit low-density lipoprotein oxidation as well as decrease the atherosclerotic process [124,125].

Xiao et al. demonstrated that green tea polyphenols alleviated the disorganized arterial wall and the increased intima-media thickness induced by a high-fat diet (HFD). The effect was also observed in the rats group fed with a standard diet. These results indicated that excess fat intakes could induce early vascular ageing (EVA) in young rats, and GTP could reverse such early vascular damage in HFD rats [126].

Talarid et al. revealed the hypertension- and glycaemia-lowering activity of polyphenols in grape-pomace-derived seasoning in subjects with a high level of cardiovascular risk. These results indicate that the reduction rate after the nutritional intervention (2 g of seasoning per day for 6 weeks) was relatively moderate. Grape pomace could also help in the control of cardiometabolic risk factors and metabolic syndrome (MetS), especially at the initial stages. [127].

Polyphenols regularly consumed in the diet may also exert anticancer properties by decreasing the growth and development of various types of cancer. Ávila-Gálvez et al. have attempted the metabolic profiling of isoflavones, lignans, and curcuminoids for the first time in breast tissues from breast cancer (BC) patients. Their results demonstrated that a mixture of polyphenolic metabolites reached the mammary tissues (MTs) from BC patients and suggest that some metabolites, especially the free curcumin occurring in MT, might exert anticancer activity after long-term exposure. The obtained results indicated that the consumption of high amounts of polyphenols could improve the glucuronidation reaction in terms of its saturation, which could allow some dietary polyphenols to reach systemic tissues in their free, much more bioactive form [128].

Polyphenols are often used to support the treatment of chronic non-communicable diseases such as diabetes and neurodegenerative diseases whose pathophysiology involves oxidative stress (OS) and inflammation [129]. Resveratrol—a polyphenol belonging to stilbenes—has proven antioxidative and anti-inflammatory activities that are attributed to its ability to activate sirtuin 1 (SIRT1), and, consequently, this activation stimulates AMP-dependent protein kinase (AMPK), which manages to improve biogenesis and mitochondrial function, increase insulin sensitivity, attenuate oxidative damage, and regulate metabolic homeostasis. In some studies on animal models with T2DM, resveratrol exerted antioxidant, anti-inflammatory, and even hypoglycaemic effects [130]. In this context, García-Martínez et al. demonstrated in a randomized clinical trial on adults with T2D that supplementation with resveratrol (with two doses: 1000 mg/day and 500 mg/day) resulted in an increase in the total antioxidant capacity, antioxidant gap, percentage of subjects without oxidant stress, and sirtuin 1 levels in comparison to those of the controls. Moreover, the observed antioxidant effect of resveratrol was more noticeable for the 1000 mg dose [131].

In the context of T2DM, Grabežet al. conducted a prospective, randomized, double-blind, placebo-controlled trial of the effect of polyphenols in pomegranate peel extract (PoPEx) on the outcomes of inflammatory factors and oxidative stress in patients with type 2 diabetes mellitus. Patients were randomly assigned to one of two groups: the first (n = 30) received PoPExin 250 mg capsules twice a day, while the second (n = 30) received placebo capsules twice a day. The plasma levels of inflammatory factors (IL-6, TNF-, and high-sensitivity C reactive protein (hsCRP)), oxidative stress biomarkers (thiobarbituric acid reactive substances (TBARS)), nitrites (NO_2_^−^), the superoxide anion radical (O_2_•^−^), hydrogen peroxide (H_2_O_2_), the total antioxidant capacity (TAC), homocysteine, and the lipid profile were measured. The PoPEx therapy reduced inflammatory factors (IL-6, TNF-, hsCRP), oxidative stress biomarkers (TBARS, NO_2_^−^, O_2_•^−^), and homocysteine, while increasing TAC. Furthermore, the PoPEx group showed a significant improvement in lipid profile. In the PoPEx group, there was a significant opposite relationship between the decreases in all the determined inflammatory markers and TAC [132].

Polyphenols belonging to various groups have also demonstrated neuroprotective and anti-ageing properties. Their activity in the brain encompasses the prevention of neuronal fatty acids’ oxidation, a reduction in the damage caused by reactive oxygen and nitrogen species, and an improvement in neurocognition by facilitating de novo protein synthesis in key sites and neurogenesis in the dentate gyrus [133]. For example, Vina et al. found that patients with Alzheimer’s disease supplemented with genistein at a dose of 120 mg/day for 12 months exhibited a significant improvement in two of the tests used (dichotomized direct TAVEC and dichotomized delayed Centil REY copy). In genistein-treated patients, the amyloid-beta deposition analysis revealed that they did not increase their uptake in the anterior cingulate gyrus after treatment as opposed to the placebo-treated group for which an increase was noted [134]. This study demonstrated that genistein may have a therapeutical role in and may have delayed the onset of Alzheimer’s dementia in patients with prodromal Alzheimer’s disease. In another study, Boespflug et al. showed that in older adults with cognitive impairment, blueberry powder (with an anthocyanin concentration near 15 mg) supplementation improved their neuronal responses, observed by magnetic resonance imaging (MRI) during memory challenges [135].

## 6. Metabolism of Polyphenols:

The bioavailability of polyphenols can be influenced by multiple factors such as the food matrix, processing methods, and individual variations in metabolism. Some polyphenols may have low bioavailability and thus a significantly low absorption or rapid metabolism. It needs to be emphasized that polyphenols present poor absorption in the small intestine, and their bioactivity in living organisms is also generally strictly correlated with the activity of the microbiota [136]. Nevertheless, even if their systemic concentration is relatively low, polyphenols can still exert local effects on the gastrointestinal tract, contributing to gut health and microbiota [137]. Polyphenols undergo various biotransformation processes, including phase I and phase II reactions. Phase I reactions involve the conversion of polyphenols by enzymes such as cytochrome P450, resulting in the formation of intermediate metabolites. These reactions include hydroxylation, demethylation, dehydroxylation, and oxidation. The resulting products are intermediate metabolites, which can exhibit different biological activities compared to the parent compounds. Phase II reactions include conjugation with endogenous compounds, such as glucuronic acid, sulphate, or methyl groups, leading to water-soluble metabolites that can be readily excreted. The gut microbiota also plays a crucial role in the metabolism of polyphenols, generating metabolites with potential biological activities. Polyphenols that escape metabolism in the upper gastrointestinal tract reach the large intestine, where they undergo extensive microbial metabolism by the gut microbiota. The gut microbiota can enzymatically convert polyphenols into smaller phenolic acids and other metabolites. These microbial metabolites can have unique biological activities and contribute to the overall health benefits associated with polyphenol consumption [138].

The daily consumption of polyphenols has been estimated to be 900 mg [139]. This level of intake is ten times that of vitamin C and one hundred times that of vitamin E or b-carotene [137]. Several factors, however, make an accurate calculation of polyphenol intake hard: (1) Polyphenols have an extensive variety of chemical structures. (2) Polyphenols are typically found in a wide range of foods. Some polyphenols, such as quercetin, can be found in a number of foods, whereas others, such as flavanones in citrus, are often specific to a particular species of plant or plant family. (3) The amount of each in a specific food can vary greatly as a result of a variety of factors. (4) There are no standard procedures for determining polyphenols in foods or methods of quantification. Catechins and proanthocyanidins are the most abundant flavonoids in the human diet, accounting for roughly three-quarters of the total flavonoids consumed. Phenolic acids make up a large portion of the polyphenols consumed by coffee drinkers. Estimates of total polyphenol intake are clearly affected by the number of polyphenol classes or subclasses included in the survey, particularly the inclusion of proanthocyanidins and phenolic acids. Few authors have included these two final polyphenol groups.

Several studies have investigated polyphenol intake in different populations by using 24-hour recalls or semi-quantitative food frequency questionnaires and polyphenols databases [140,141,142,143]. For example, the average daily intake of polyphenols in the Spanish diet is around 3000 mg/person/day [140], although the PREDIMED research of 7000 Spanish people found their total phenols intake to be 820 ± 323 mg per day, with flavonoids and phenolic acids being the most abundant components [142]. In France, the total polyphenol intake in 4000 adults is estimated at 1193 ± 510 mg/d [141] and in Poland, it is estimated at 1756.5 ± 695.8 mg/d [143].

Despite an increasing body of evidence for polyphenol bioavailability in the systemic circulation [144], little is known about their ability to reach the central nervous system (CNS). Animal studies show that polyphenols can cross the blood–brain barrier (BBB) and colocalize in tissues of the brain despite the route of administration. For example, naringenin was discovered in the brain after the injection [145], whereas epigallocatechin gallate [146], epicatechin [147], and anthocyanins [148,149] were identified after swallowing. Schaffer et al. demonstrated that polyphenols usually localize in the brain at levels below 1 nmol/g tissue [150]. Polyphenols and their degraded metabolites can also aid in the treatment of T2D. These components converted into secondary metabolites by certain gut microbiota have higher absorption and bioactivity than their precursors [151]. The flavonoid metabolites 3,4-dihydroxyphenylacetic acid and 3-hydroxyphenylpropionic acid which are derived from microbes may have anti-diabetic properties through encouraging the growth and functioning of pancreatic cells [152]. Furthermore, polyphenol microbial metabolites could control bile acid production, which impacts the metabolism of the host.

It is worth mentioning that the cellular environment and/or concentration of polyphenols is a factor with a diverse influence on the form of the displayed activity of polyphenols, either antioxidants or pro-oxidants [153]. Due to their pro-oxidant properties, polyphenols can have toxicological effects when consumed in high concentrations [154,155]. Their potential detrimental effects on health can be attributed to their capacity to activate transcription factors, such as NF-B, activating protein-1 (AP-1), tumour protein (p53), and Nrf2, as well as cause cell damage through the collapse of the mitochondrial membrane potential, favouring the propagation of reactive species and subsequently the inflammatory process [156].

In this regard, Moridani and colleagues evaluated the toxicity of flavonoids in isolated rat hepatocytes and in HeLa tumour cells. They discovered that epicatechin had the lowest toxicity (LD50 = 17,000 µmol) and that galangin had the highest toxicity after 2 h, with lipophilicity being the primary determinant of the cytotoxicity of the evaluated polyphenols [157]. According to a study by Chen et al. [158], genistein consumption at high concentrations (200 µmol) increased the production of reactive oxygen species (ROS) in a way that was dependent on 5-lipoxygenase, an essential enzyme in the biosynthesis of leukotrienes, which in turn plays a crucial role in the inflammatory response. Furthermore, high dietary doses of green tea polyphenols (0.5–10%) also worsen colitis and colon carcinogenesis in ICR mice and cause nephrotoxicity, hepatotoxicity, and the negative regulation of the expression of antioxidant enzymes and molecular chaperones [159]. Since phenolic compounds can adversely affect health depending on the concentration used, careful consideration must be given to their intake [156].

### 6.1. Mechanisms of Antidiabetic Activity of Phenolics

The inhibition of carbohydrate digestion by salivary and pancreatic α-amylase and α-glucosidase inhibition in the small intestine, glucose absorption inhibition, and insulin secretion stimulation and protective activity towards pancreatic β-cells against glucotoxicity damage are indicated as potential mechanisms of the hypoglycaemic action of polyphenolic compounds. The effects of the suppression of gluconeogenesis in the liver and increase in glucose uptake in peripheral tissues on the modulation of intracellular signalling pathways are other proposed mechanisms of polyphenols’ activity [160]. De Paulo Farias et al. [161] presented in their review the antidiabetic effects of dietary phenolic compounds and their mechanisms of action. It was discovered that these compounds have a high therapeutic potential for diabetes management, and their mode of action includes several mechanisms related to oxidative stress reduction, the inhibition of DPP-IV and enzymes involved in carbohydrate metabolism, a reduction in insulin resistance, and AGE formation, among others. Nevertheless, it is necessary to note that the anti-diabetic properties of phenolic compounds largely depend on factors such as food concentration, bioaccessibility, absorption, metabolism, and bioavailability, indicating the need for research into the relationship of these factors with phenolic bioactivity to outline strategies aimed at maximizing its effects on diabetes management. Moreover, care should be taken to establish safe doses, as these compounds can have negative health effects when consumed in large quantities.

**Figure 6:**
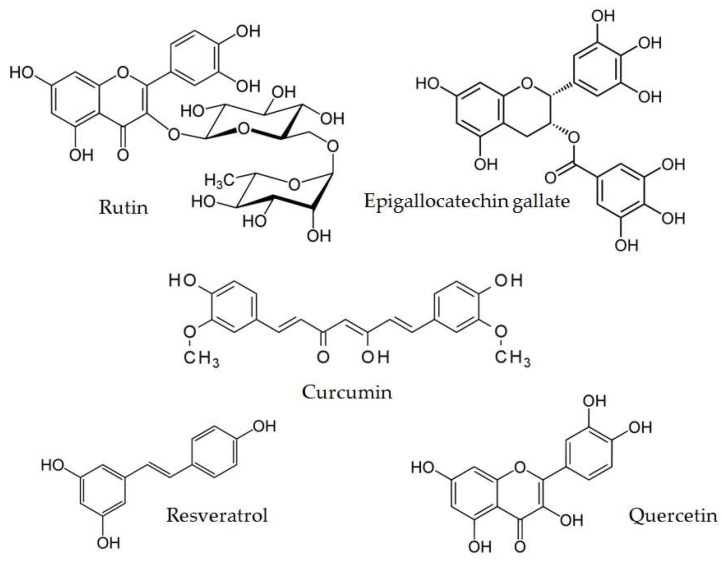
Structures of example polyphenols.

Next sections of this paper are presenting some of the already revealed antidiabetic activities of most investigated phenolic compounds, the chemical structures of mentioned compounds are presented in Figure 6.

### 6.2. Animal Studies on AntidiabeticEffects of Polyphenols

Many studies have used diabetic streptozotocin (STZ) models or leptin-deficient *db*/*db* mice. Although in a mechanistic manner, some of the applied models seems to be more suitable for the investigation of T1DM than T2DM. Nonetheless, they provide helpful information on some of the numerous anti-diabetic properties of polyphenolic compounds.

Do et al. [162] studied resveratrol supplementation in *db*/*db* mice, and they concluded that this famous stilbene decreased blood glucose and HbA1c levels. Simultaneously, increased plasma and pancreatic insulin content was observed. It could suggest that resveratrol improves glucose tolerance and β-cell mass preservation [163].

Curcumin administration to rats fed a high-fat diet (HFD) and STZ resulted in lower fasting blood glucose levels, increased insulin levels, and insulin sensitivity [164]. Curcumin treatment reduced blood glucose and Hb1Ac levels in alloxan-induced diabetic rats and db/db mice [165]. Curcumin has also been shown to improve insulin resistance and glucose tolerance in db/db mice and HFD-fed mice [166], as well as to increase insulin levels in db/db mice. Curcumin supplementation did not, however, affect blood glucose levels in a similar study of db/db mice [167].

Studies on treating animals with green tea demonstrated that it could prevent hyperglycaemia, hyperinsulinemia, and insulin resistance in a high-fructose diet [168]. Additionally, it significantly reduced the amount of glucose in the blood of STZ-diabetic and *db*/*db* diabetic mice; nevertheless, it exhibited no effect on serum insulin levels [169]. However, some studies also demonstrated that particular green tea components showed inconsistent results. In the case of Epigallocatechin gallate (EGCG), its antidiabetic effects depend on the way of its administration. In STZ-treated mice, an intraperitoneal injection of EGCG decreased hyperglycaemia while preserving islet mass [170]. After 4 weeks, oral EGCG showed no effect on blood glucose levels in mice fed a HFD [171]. On the other hand, long-term oral dosing has been shown to reduce blood glucose in *db*/*db* mice, non-obese diabetic mice, and STZ rats [172].

Both rutin and its aglycone derivative compound, quercetin, also show interesting antidiabetic properties. STZ-rats treated orally with rutin showed decreased plasma glucose levels and increased insulin levels [173], and the C-peptide concentration was also increased [174]. Quercetin additionally protected β-cells against STZ-activated degeneration [175].

### 6.3. Human Studies on Antidiabetic Effects of Polyphenols

Ultimately, pre-clinical data have confirmed polyphenols as chemicals having metabolic regulation effects that may help to prevent or postpone the onset of type 2 diabetes, although human evidence is still lacking. There have only been a few meta-analyses of randomized controlled human studies examining the impact of polyphenols on diabetes biomarkers or incidence, and some of them have shown confusing results. For example, in one meta-analysis, biomarkers such as blood glucose, fasting insulin, or HbA1c were reported to be affected by a specific polyphenol but not another [176,177,178].

Many experiments with green tea and similar catechins have been conducted, but the findings have not been consistent. Zheng et al. [178] performed a meta-analysis to identify and quantify the effects of green tea catechins and green tea catechins’ caffeine mixture on the glucose metabolism of adults. These studies showed that green tea catechins, with or without caffeine, substantially lowered fast blood glucose levels but had no effect on fast blood insulin, HbA1c, or the homeostatic model assessment of insulin resistance (HOMA-IR). A subgroup analysis revealed that the glucose-lowering impact was more pronounced when the follow-up period exceeded a median of 12 weeks. Green tea doses, intervention type (with or without caffeine), research quality, ethnicity, habitual caffeine intake, and participant health status did not appear to significantly impact the pooled mean differences in the fast blood glucose or fast blood insulin concentrations. Another meta-analysis of green tea’s effect on individuals with T2DM or prediabetes revealed no effect on the fasting blood glucose, HbA1c, insulin, or HOMA-IR. However, these findings may be less credible due to the small sample numbers and the diverse quality of the research evaluated [179].

In turn, Rienks et al. [180] evaluated polyphenol exposure and risk of T2DM development and demonstrated that diets high in polyphenols, particularly flavonoids, can help avoid type 2 diabetes. Evidence of nonlinearity was discovered for most relationships, indicating a recommended level of consumption linked with the lowest risk of type 2 diabetes.

Human research on resveratrol has likewise had inconsistent results. Four weeks of resveratrol supplementation at a low dosage (10 mg/day) lowered HOMA-IR in T2DM patients but did not affect blood insulin levels or βß-cell function indicators [181]. In contrast, 12 weeks of a significantly larger dosage (3 g/day) in participants with T2DM did not improve HOMA-IR, despite a nonsignificant drop in HbA1c [182]. In contrast, Timmers et al. [183] discovered that 30 days of 150 mg/day resveratrol lowered levels of fasting plasma glucose, insulin, triglycerides, and HOMA-IR. Further follow-up trials have also failed to provide any significant differences. A 6-month experiment in T2DM patients exhibited no difference in levels of blood glucose, HbA1c, insulin, C-peptide, HOMA-IR, or FFA [184].

Studies on curcumin are much more promising. Some evidence has suggested that 12 weeks of therapy, including curcumin in participants with a high risk of T2DM development, caused decreased levels of plasma insulin with no effect on glucose levels [185]. Curcuminoid medication for three months lowered fasting blood glucose, HbA1c levels, and insulin resistance in individuals diagnosed with T2DM or prediabetes [186].

Ostadmohammadi et al. [187] conducted a systematic review to determine the effect of quercetin supplementation on glycaemic control among patients with metabolic syndrome (MetS) and related disorders. A meta-analysis found that quercetin supplementation did not affect glycaemic control in people with MetS and other associated illnesses. A subgroup study based on an 8-week-long trial with quercetin doses of 500 mg/day significantly lowered FPG levels. In addition, in trials with participants aged 45 years and quercetin doses of 500 mg/day, their insulin levels decreased considerably after quercetin supplementation.

More detailed data revealed in all described above studies are presented in the Table 1.

In conclusion, considering the significant attention and quantity of randomized controlled trials investigating the influence of polyphenols on metabolic- or diabetes-related outcomes, the results remain disputed. Inconsistent findings may be attributable to discrepancies in terms of the sample dose, time frame, health status of individuals, whether participants were taking other medications, and the small sample sizes frequently used. It needs to be emphasized that outcomes are not always comparable with a treatment’s dose or time-dependent impact. The data show that polyphenol therapies may help decrease blood glucose and maybe improve insulin sensitivity, although more and better research is needed. Natural polyphenols have a favourable safety profile and are found in relatively high concentrations in the average human diet.

## 7. Conclusions

This review focused on the anti-diabetic properties of dietary phenolic compounds and their mechanisms of action. Nevertheless, it is essential to note that the final anti-diabetic activity of phenolic compounds is dependent on numerous factors, including their concentration in food, absorption, metabolism, and bioavailability, indicating the need for research into the association of these factors with phenolic bioactivity to outline strategies aimed at maximizing the impact on diabetes management. Therefore, given the importance of diabetes and the rising number of people suffering from it, there is a clear need for novel medicines that may lessen the detrimental effects of the condition while also ensuring the safety and well-being of the community. In this regard, polyphenols might be a possible option for controlling the course of this metabolic disease.

Presented in this review, numerous in vitro and in vivo studies have been selected from copious, currently ongoing, or recently completed studies and trials. They all indicate that a diet rich in polyphenolic compounds benefits glucose homeostasis through multiple and complex mechanisms of action in various human body organs such as the intestine, liver, muscle, adipocytes, and pancreatic β-cells. Polyphenol intake seems to be associated with a lower T2DM development and progression rate. The clinical studies that have been conducted have shown significant limitations and inconsistencies when considering dietary polyphenol supplementation. However, applying these compounds in glucose and insulin homeostasis management seems to be the right direction and a promising approach for further studies and research.

Due to the poor bioavailability of polyphenols, progress in micro- and nanoencapsulation methodologies of phenolic compounds could be applied as supportive supplements for diabetes treatment. The encapsulation of phenolic compounds may additionally cover the unpleasant flavour of some plant extracts, paving the way for the development of future functional foods, nutraceuticals, or orally administered polyphenol-based diabetes drugs.

## Figures and Tables

**Table 1:** Details of antidiabetic activities revealed in reported studies.

Investigated Phenolic Compounds and Their Origin	Experimental Design	Revealed Antidiabetic Activities	Ref.
**Animal Model Studies In Vitro and In Vivo**			
**Resveratrol** (3,5,4′-trihydroxy-trans-stilbene) Calss: Stilbenoid	C57BL/KsJ-db/db mice fed with a normal diet with RV (0.005% and 0.02%, *w*/*w*) or rosiglitazone (0.001%, *w*/*w*) for 6 weeks	↓blood glucose ↓plasma free fatty acid and triglyceride, ↓apo B/apo AI levels ↓HbA1c levels ↓hepatic gluconeogenic enzyme activity and hepatic glycogen ↓hepatic triglyceride content and p-IKK protein expression ↑plasma insulin levels ↑pancreatic insulin protein ↑skeletal muscle GLUT4 protein ↑plasma adiponectin levels ↑hepatic glycolytic gene expression and enzyme activity ↑skeletal muscle glycogen synthase protein expression ↑hepatic UCP and skeletal muscle UCP expression	[162]
	db/db and db/dm mice (non-diabetic control) were treated with or without RV (20 mg/kg BW daily) for 12 weeks	↑glucose tolerance at 2 h of OGTT in db/db mice ↑pancreas weight and β-cell mass ↓urinary 8-OHdG levels ↓percentage of islet nuclei that were positive for 8-OHdG immunostaining	[163]
**Curcumin** (1E,6E)-1,7-Bis(4-hydroxy-3-methoxyphenyl)hepta-1,6-diene-3,5-dione) Class: Curcuminoid	diabetic rats induced by high-fat diet plus STZ (30 mg/kg BW) were fed a diet containing 50, 150, or 250 mg/kg BW curcumin for 7 wks	↓plasma lipids and glucose ↓glucose and insulin tolerance ↓pyruvate dehydrogenase 4 and GS expression ↑2-deoxy-[(3)H]d-glucose uptake by L6 myotubes ↑phosphorylated AMPK, CD36, and carnitine palmitoyl transferase 1 expression ↑phosphorylated acetyl COA carboxylase in L6 myotubes	[164]
	male C57BL/KsJ-db/db mice and their age-matched lean non-diabetic db/+ mice, fed with or without curcumin (0.02%, *w*/*w*) for 6 weeks	↓blood glucose and HbA 1c levels, as well as body weight loss ↓glucose-6-phosphatase and phosphoenolpyruvate carboxykinase activities ↓hepatic activities of fatty acid synthase, beta-oxidation, 3-hydroxy-3-methylglutaryl coenzyme reductase, and acyl-CoA: cholesterol acyltransferase ↓plasma free fatty acid, cholesterol, and triglyceride concentrations ↑homeostasis model assessment of insulin resistance and glucose tolerance ↑plasma insulin level ↑hepatic glucokinase activity ↑hepatic glycogen and skeletal muscle lipoprotein lipase	[165]
	male C57BL/6J mice were fed either a normal diet or HFD.After 16 weeks, 10 HFD-fed mice were further treated with daily curcumin oral gavage (50 mg/kg BW)	↓glucose intolerance ↓HFD-induced elevations of MDA and ROS in the skeletal muscle ↑skeletal muscle content of Nrf2 and oxygenase-1	[166]
	db/db livers of 15-week-old mice treated with 0.75% curcumin mixed in their diet for 8 weeks	↑expression of AMPK and PPARγ ↓NF-κB protein levels	[167]
**Green tea polyphenols** (mixture)	SpragueDawley rats fed with standard chow and deionized distilled water and a “green tea” group fed the same chow diet but with green tea instead of water (0.5 g of lyophilized green tea powder dissolved in 100 mL of deionized distilled water) for 12 weeks	↓fasting plasma levels of glucose, insulin, triglycerides, and free fatty acids ↑insulin-stimulated glucose uptake and insulin binding; adipocytes were significantly increased ↑basal and insulin-stimulated glucose uptake of adipocytes in vitro	[168]
	C57BLKS/J *db+/db+* mice and age-matched control C57BLKS/J +m/+m mice. Male ddY mice were singly injected with STZ (150 mg/kg, i.v.)and 4–6 weeks after the injection the samples were analyzed.The age-matched normal ddY mice were also used	↓blood glucose levels in diabetic db+/db+ mice and streptozotocin-diabetic mice 2–6 h after administration at 300 mg/kg 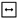 serum insulin level	[169]
**Epigallocatechin gallate** ((EGCG), (2R,3R)-3′,4′,5,5′,7-Pentahydroxyflavan-3-yl 3,4,5-trihydroxybenzoate) Class: Catechin	maleC57BL/KsJ mice aged 6 weeks induced with multiple low doses of streptozotocin (MLD-STZ, (40 mg/kg BW). EGCG (100 mg/kg/day) was administered with STZ for 5 days and then EGCG alone was administered for a further 5 days	↓blood glucose levels ↓iNOS expression ex vivo ↓decrease in islet mass induced by MLD-STZ	[170]
**Epigallocatechin gallate continued**	C57BL/6 male mice (6 weeks old) were fed with ND, HFD, or HFD with EGCG supplementation for 12 weeks. EGCG (50 mg/kg daily) was administered by gavage for 9–12 weeks	Inhibition of Caspase-1 activation and IL-1β secretion in mice bone marrow by suppressing NLRP3 inflammasome activation Inhibition of NLRP3-mediated ASC speckle formation and alleviated pyroptosis in BMDMs Improved high-fat-diet (HFD)-induced glucose tolerance	[171]
	male albino Wistar rats induced with by a single i.p. injection of 60mg/kg^−1^STZ received EGCG 25mg/kg/day for 8 weeks 1 week after the induction of diabetes	Hypoglycaemic effect in diabetic rats Improved serum lipid profile Attenuation of increased MDA content and reduced activity of SOD in liver.	[172]
**Rutin** (3′,4′,5,7-Tetrahydroxy-3-[α-L-rhamnopyranosyl-(1→6)-β-D-glucopyranosyloxy]flavones) Class: Flavonoid glycoside	adult male SpragueDawley rats injected withSTZ i.p. (55mg/kg BW) after induction ofdiabetic neuropathy.Rutin (5mg/kg, 25mg/kg and 50mg/kg BW) was daily given to the diabetic rats for 2 weeks	Inhibition of mechanical hyperalgesia, thermal hyperalgesia, and coldallodynia ↑Na^+^, K^+^-ATPase activities insciatic nerves ↑hydrogen sulfide(H_2_S) level, upregulated expression of nuclear factor-E2-related factor-2 (Nrf2), and heme oxygenase-1 (HO-1) in DRG ↓caspase-3 expression indorsal root ganglions ↓plasma glucose, attenuatedoxidative stress,andneuroinflammation Partial restoration ofnerve conductionvelocities in diabetic rats	[173]
	male albino Wistar rats induced with i.p. injection of STZ (50 mg/kg BW).Rutin (25, 50, 100 mg/kg BW) was orally administered to normal and diabetic rats (1 mL/rat) using an intragastric tube for a period of 45 days	↑fasting plasma glucose, HbA1c, thiobarbituric acid reactive substances, and lipid hydroperoxides ↓blood insulin, C-peptide, total haemoglobin, protein levels, non-enzymic antioxidants: glutathione, vitamin C, vitamin E, and ceruloplasmin	[174]
**Quercetin** (3,3′,4′,5,7-Pentahydroxyflavone) Class: Falvonoid	Male adult albino Wistar rats induced by a single injection of STZ (45 mg/kg, i.p.). Diabetic rats were orally treated with sitagliptin (70 mg/kg BW), quercetin (50 mg/kg BW), or a combination of these daily for 3 weeks	↑increased SOD, GSH ↓NF-κB expression Normalized Islet number, β-cells’ number, area, and perimeter alongside the restoration of the immunostaining intensity of β-cells.	[175]
**Human studies**			
**Green tea polyphenols** (mixture)	A total of 17 trials comprising a total of 1133 subjects were included in the current meta-analysis	↓fasting blood glucose ↓Hb A1c ↓fasting blood insulin	[188]
**Green tea catechins** (mixture)	A total of 22 eligible randomized controlled trials with 1584 subjects were identified	↓fasting blood glucose 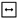 Hb A1c 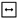 fasting blood insulin 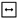 HOMA-IR	[189]
**Green tea** (mixture and extracts)	A total of six studies with 382 subjects were pooled into random-effects meta-analysis	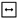 HOMA-IR 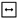 HbA1c 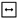 fasting blood insulin 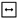 fasting blood glucose	[176]
**Polyphenols** (mixture in supplements and food)	A total of 36 controlled randomized trials with 1954 subjects were included in 28 mg to 1.5 g of polyphenol mixture, supplemented for 0.7 to 12 months	↓HbA1c in T2DM individuals	[179]
**Polyphenols** (51 different compounds in Total)	A total of 18 studies investigated the association between polyphenols and type 2 diabetes	Evidence showing that diets rich in polyphenols, and particularly flavonoids, play a role in the prevention of type 2 diabetes.	[180]
**Resveratrol** (3,5,4′-trihydroxy-trans-stilbene) Calss: Stilbenoid	A total of 19 patients enrolled in the 4-week-long double-blind study were randomly assigned into two groups: an RV group receiving oral 2 × 5 mg RV and a control group receiving placebo	↓HOMA-IR ↓urinary ortho-tyrosine excretion ↑pAkt:Akt ratio in platelets	[181]
	Ten subjects with T2DM were randomized in a double-blind fashion to receive 3 g RV or placebo daily for 12 weeks	↑SIRT1 expression ↑pAMPK to AMPK expression ratio ↓average daily activity ↓step counts	[182]
	A total of 11 healthy and obese men received placebo and 150 mg/day RV in a randomized double-blind crossover study for 30 days	↓sleeping and resting metabolic rate ↓intrahepatic lipid content ↓circulating glucose, triglycerides, and alanine-aminotransferase ↓inflammation markers ↓systolic blood presure ↑AMPK activity ↑SIRT1 and PGC-1α protein levels ↑citrate synthase activity ↑intramyocellular lipid levels Improved muscle mitochondrial respiration on a fatty acid-derived substrate and HOMA-IR index	[183]
**Resveratrol** continued	double-blind, randomized, placebo-controlled trial, with192 T2DM patients randomized to receive RV 500mg/day, 40mg/day, or placebo for 6months	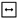 weight, BMI, waist circumference 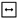 arterial blood pressure 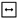 fasting glucose, plasma insulin, C-peptide, free fatty acids, liver transaminases, uric acid, adiponectin, and interleukin-6 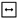 HbA1c	[184]
**Curcumin** (1E,6E)-1,7-Bis(4-hydroxy-3-methoxyphenyl)hepta-1,6-diene-3,5-dione) Class: Curcuminoid	Plasma samples from 29 participants recruited for a randomised controlled trial with curcumin (180 mg/day) for 12 weeks were analysed	↓Levels of circulating GSK-3β and IAPP ↓insulin resistance	[190]
**Curcumin + Zinc**	A total of 84 subjects were randomized into curcumin (500 mg), zinc (30 mg), zinc and curcumin, and placebo groups for 90 days	↓BMI Improved fasting blood glucose, HbA1c, blood insulin, and HOMA-IR	[186]
**Quercetin** (3,3′,4′,5,7-Pentahydroxyflavone) Class: Falvonoid	A total of nine studies of 781 participants involved in meta-analysis	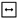 fasting plasma glucose, HOMA-IR, HbA1c ↓fasting blood glucose in studies with a duration of ≥8 weeks ↓insulin concentrations in studies that enrolled individuals aged <45 years	[187]
